# Machine Learning Modeling of *Aedes albopictus* Habitat Suitability in the 21st Century

**DOI:** 10.3390/insects14050447

**Published:** 2023-05-09

**Authors:** Pantelis Georgiades, Yiannis Proestos, Jos Lelieveld, Kamil Erguler

**Affiliations:** 1 Environmental Predictions Department, Climate and Atmosphere Research Centre, Cyprus Institute, 2121 Nicosia, Cyprus; y.proestos@cyi.ac.cy (Y.P.); jos.lelieveld@mpic.de (J.L.); 2 Computation-Based Science and Technology Research Center (CaSToRC), Cyprus Institute, 2121 Nicosia, Cyprus; 3 Max Planck Institute for Chemistry, Hahm-Meitner-Weg 1, 55128 Mainz, Germany

**Keywords:** machine learning, vector-borne diseases, habitat suitability

## Abstract

**Simple Summary:**

The Asian tiger mosquito, *Aedes albopictus*, is a highly invasive and adaptive vector of viruses that can cause human diseases, such as dengue, chikungunya, and zika. As climate and socio-economic changes continue, the mosquito’s suitable habitat range is expected to expand, posing a significant threat to global public health. To predict the shifts in the mosquito’s global habitat suitability, we developed an ensemble machine learning model that combines a Random Forest and XGBoost binary classifiers. The model was trained using global vector surveillance data and a collection of climate and environmental constraints. We project a significant expansion of the mosquito’s habitat suitability, with at least an additional billion people at risk of vector-borne diseases by the mid-21st century. A number of highly populated areas of the world, such as the northern parts of the USA, Europe, and India, will be at risk of *Ae. albopictus*-borne diseases by the end of the century. Our findings highlight the need for coordinated preventive surveillance efforts by local authorities and stakeholders to control the spread of the mosquito and prevent disease outbreaks.

**Abstract:**

The Asian tiger mosquito, *Aedes albopictus*, is an important vector of arboviruses that cause diseases such as dengue, chikungunya, and zika. The vector is highly invasive and adapted to survive in temperate northern territories outside its native tropical and sub-tropical range. Climate and socio-economic change are expected to facilitate its range expansion and exacerbate the global vector-borne disease burden. To project shifts in the global habitat suitability of the vector, we developed an ensemble machine learning model, incorporating a combination of a Random Forest and XGBoost binary classifiers, trained with a global collection of vector surveillance data and an extensive set of climate and environmental constraints. We demonstrate the reliable performance and wide applicability of the ensemble model in comparison to the known global presence of the vector, and project that suitable habitats will expand globally, most significantly in the northern hemisphere, putting at least an additional billion people at risk of vector-borne diseases by the middle of the 21st century. We project several highly populated areas of the world will be suitable for *Ae. albopictus* populations, such as the northern parts of the USA, Europe, and India by the end of the century, which highlights the need for coordinated preventive surveillance efforts of potential entry points by local authorities and stakeholders.

## 1. Introduction

Mosquitoes are among the most important vectors of Arthropod-borne viruses (arboviruses), which have been a public health risk throughout human history [1,2]. According to the World Health Organisation (WHO), vector-borne diseases account for more than 17% of all infectious diseases, putting more than half of the world’s population at risk. These include dengue, zika, yellow fever, and chikungunya, which can be fatal for humans and cause a significant economical and social burden. The Asian tiger mosquito, *Aedes albopictus* (Skuse, 1894) (Diptera:Culicidae), stands out as a particularly competent vector of disease due to its wide geographical distribution, resilience, and aggressiveness, and has been responsible for a number of outbreaks in the recent past [3].

*Ae. albopictus* is native in Southeast Asia, but has invaded the Americas, the Middle East and a number of European Mediterranean countries [4,5]. In view of the geographical expansion of the mosquito, the attribute “Asian” seems no longer accurate. In a number of cases in Europe, the introduction of the species is attributed to the transportation of eggs in tyres from overseas through ship trading [6]. The species exhibits a high degree of ecological plasticity, enabling it to thrive in diverse habitats and establish sustainable populations. Additionally, it has been observed to successfully reproduce in anthropogenic water receptacles in urban areas [7,8]. The temperate strain of the species has developed the ability to lay diapausing eggs, which promotes its survival in relatively cooler climates and effectively maintains its population [9]. This mosquito species is known to be highly invasive [10] and is associated with diseases that range in severity, from temporary incapacitation to premature death. Dengue is identified as the most severe disease transmitted by *Ae. albopictus*, with around forty thousand deaths attributed to it in 2017 [11]. While Zika and chikungunya are considered milder diseases, with a significantly lower death rate, they are still important as they can have long-lasting symptoms, such as joint-pain, and even be transmitted to fetuses via pregnancy [3]. As more people are exposed to vector-borne diseases [12], the potential public health and socio-economic burden may become severe [13].

With the expansion of global air and marine transportation networks, tourism and trade play a central role in the introduction (and reintroduction) of invasive vector species to countries that have the suitable habitat for population establishment [14]. Given the introduction of vectors by marine and air traffic, climate conditions can play a key role in whether the mosquitoes become endemic. Phase six of the Coupled Model Intercomparison Project (CMIP6) of the World Climate Research Program, projects a global average temperature increase of 3.22 ${}^{\circ }$C/100 yrand 7.20 ${}^{\circ }$C/100 yr by the end of the century for the Representative Concentration Pathway (RCP) 4.5 (SSP2, hereafter SSP245) and 8.5 (SSP5, hereafter SSP585) scenarios, respectively [15]. Further, precipitation is projected to decrease in the Mediterranean area, Oceania, and Central and South America, and increase in other regions of the planet [16]. The suitable periods that accommodate the reproduction and survival of many mosquito vectors, such as the *Aedes*, *Culex* and *Anopheles* genuses, are expected to increase [17], and a polewards shift of habitats appropriate for vector establishment is anticipated due to climate change [12,18,19,20].

The rapid progression of computing performance in the past two decades and the need for highly optimized and efficient automated systems have resulted in an unprecedented development in the field of machine learning and artificial intelligence [21]. This development is also reflected in the proliferation of the use of correlative environmental niche models (ENMs) [22], where machine learning methods are used to quantitatively associate species occurrence with environmental conditions and thus predict geographic spread and potential future presence.

Several methodologies have been previously employed to study and predict the geographic extend of the species’ habitat and its future potential distributional changes. Benedict et al. (2007) used a Genetic Algorithm for Rule Set Production (GARP) model, to determine the ecological niche of *Ae. albopictus* and predict a global ecological risk map for the continued spread of the species [23]. Jia et al. (2016) developed a climate-driven mechanistic population model of *Ae. albopictus* that accounts for the biological phenomenon of diapause [24]. In another study, Kamal et al. (2018) used an ecological niche modeling approach to estimate the potential distributions of *Aedes aegypti* and *Ae. albopictus* under present-day and future climate conditions [25]. The authors used occurrence records of each species and environmental variables to fit ecological niche models. Ibáñez-Justicia et al. (2020) developed habitat suitability models to investigate the potential risk of establishment and spread of *Ae. albopictus* in the Netherlands. The authors used two methodologies: first, a species distribution model based on the maximum entropy modeling approach (MaxEnt) taking into consideration updated occurrence data of the species in Europe, and secondly, a spatial logic conditional model based on the temperature requirements of the species and using land surface temperature data (LST model) [8]. One common limitation in previous studies has been the availability of reliable presence and absence datasets for species occurrence and limited spatial or temporal availability of reliable data [26].

Here, we develop an ensemble machine learning model with two complementary algorithms (Random Forest [27] and XGBoost [28]), and use an extensive set of historical climate and environmental drivers as well as human population density to model *Ae. albopictus* presence and predict habitat suitability under changing environmental conditions. Instead of using point presence and background/pseudo-absence data, we employed high-quality longitudinal surveillance datasets from a range of localities around the globe. On this basis, we project present and future potential habitat suitability following the two IPCC RCP scenarios, the “stabilization” scenario SSP245 and the “business-as-usual” scenario SSP585 [29,30,31,32,33].

## 2. Materials and Methods

This section is subdivided into two main parts, data and algorithms. In the first part, the composition and development of the datasets used in model training and evaluation are discussed. In the second part, the procedures followed for developing and evaluating the machine learning models are detailed.

### 2.1. Datasets for Supervised Learning

#### 2.1.1. Vector Presence/Absence Dataset

Abundance data for *Ae. albopictus* were obtained from surveillance efforts in Europe and the United States of America (USA) and converted to a binary class dataset with a monthly temporal resolution. The two classes created were 0 for absence of mosquitoes in a grid cell for one month and 1 for presence. A regular lon-lat grid of 0.25${}^{\circ }$ spatial resolution (1440 × 720 (lon-lat) grid cells) was adopted for the purpose of this study, which matches the CMIP6 dataset grid size [34]. Surveillance data were obtained from the following sources:

- The Vectorbase PopBio (MapVEu tool) database was extracted for *Ae. albopictus*, for a period spanning from 2003 to 2021 [35]. The database was queried for *Ae. albopictus* in taxonomy and the “abundance” data type. The data request to the database included zero-counts.
- *Ae. albopictus* surveillance data from the Emilia-Romagna region in Italy for the years 2008–2012 [36]. These include bi-weekly surveillance data from ovitraps placed throughout the region.
- Surveillance data from Hungary (2017–2019), Slovenia (2016), and Serbia (2018) which were kindly provided by Prof. Dušan Petrić (University of Novi Sad), Dr. Kornélia Kurucz (University of Pécs), Dr. Katja Kalan (University of Primorska), and Dr. Ognyan Mikov (National Centre of Infectious and Parasitic Diseases, Bulgaria) [37].
- Data provided for the project Aedes challenge 2019 and 2020 from the Centre of Disease Control (CDC), accessed on 10 October 2021 (https://predict.cdc.gov), for *Ae. albopictus*. These data are provided in administrative units [38].

The data from the aforementioned sources were harmonized to match the 1440 × 720 (lon-lat) global grid used for this study. Data for which the geographical position was reported in terms of longitude and latitude were cross-referenced with the mesh used to determine in which 0.25${}^{\circ }$ grid box they were located. Data which were reported in terms of administrative units, the corresponding grid cells contained within and intercepted each administrative unit were determined. The feature set (climate, land use, and population density) for the group of grid cells corresponding to each administrative unit were subsequently averaged. Consequently, the data were pooled into the form of a uniform gridded monthly presence/absence dataset.

**Figure 1:**
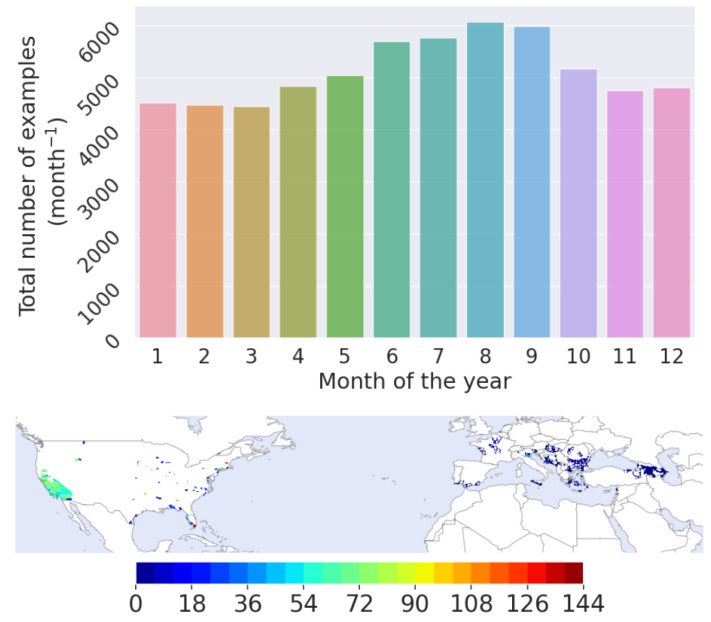
Distribution of the number of examples in the training set for each month of the year (**top** panel) and geographical distribution of the dataset used to train and evaluate the model’s performance (**bottom** panel). The colour bar shows the number of examples for each grid cell.

The geographical distribution of the dataset used for training and evaluating the performance of the machine learning model is shown in Figure 1. As shown, the vast majority of the examples originate from the USA. Grid cells in which the number of examples was less than 5 were omitted from the training dataset as well as grid cells in which only the negative class was present (i.e., no *Ae. albopictus* was detected), as it was not clear if that was due to environmental factors or simply due to the vector being absent from the region. In addition, grid cells in which surveillance data were only available during the peak season (usually summer months) and were only represented by the positive class, were also omitted to avoid adding bias to the machine learning model. As shown in Figure 1, in the bar plot representing the distribution of examples for each month of the year, the peak season is represented by a larger amount of examples compared to the winter months. There is adequate representation for every month in the year to capture longitudinal fluctuations in the grid cells, such as temperature and precipitation patterns.

#### 2.1.2. Feature Dataset

The spatio-temporal global grid (0.25${}^{\circ }$ spatial and monthly temporal resolution) was described using climate, land use, and population density to create the training dataset and to project *Ae. albopictus* habitat suitability until the end of the 21st century. In this section the datasets used and the steps performed to create the uniform feature set are described.

Land use data were obtained from the Land Use Harmonization (LUH2) program, part of the Climate Research Program Coupled Model Intercomparison (CMIP6) project [39,40]. The Shared Socioeconomic Pathways (SSP2) Representative Concentration Pathway 4.5 (SSP245) dataset was employed for the training set and predictions for the stabilization pathway scenario, whereas the SSP5 8.5 (SSP585) dataset was used for the corresponding business-as-usual scenario [15]. The spatial resolution of these datasets matched the 1440 × 720 (lon-lat) grid used in this study and is provided with an annual temporal resolution. To match the monthly temporal resolution of the vector surveillance data, we performed temporal interpolation using the xarray package in Python (nearest-neighbour method) [41].

The land use states datasets were used in this study, which denote the fraction of each grid cell occupied by the various land uses in a given year. A total of 14 classes are provided in the LUH2 land use datasets, from which the urban class was used intact and others were combined in order to produce features relevant to the vector’s habitat dependencies. The four additional features created from the land use dataset were:

- **Forested.** Created by adding the primf (primary vegetation - potential forest land) and secdf (secondary vegetation - potential forest land) classes for each grid box/month.
- **Non-forested.** Created by adding the primn (primary vegetation - potential non-forest land) and secdn (secondary vegetation - potential non-forest land) classes.
- **Crops.** Created by adding the crops related classes; c3ann (C3 annual crops), c4ann (C4 annual crops), c3per (C3 perennial crops), c4per (C4 perennial crops), and c3nfx (C3 nitrogen-fixed crops).
- **Graze land.** Created by adding the pastr (managed pasture) and range (range land) classes.

Projections for human population density for the matching SSP245 and SSP585 scenarios were obtained from Jones and O’Neil (2016) at 8 km spatial and a 10-year temporal resolution [42]. The dataset was re-gridded to match the spatial resolution of the vector surveillance dataset and temporally interpolated to the monthly (linear method) using the xarray Python package.

The photo period (time between sunrise and sunset) was calculated using the Brock model [43], defined as the point where the sun’s center is even with the horizon. To calculate the day-length, the declination of the Earth is computed using [44]:

(1) $$\phi =23.45*sin(\frac{283+J}{265})$$

where *J* is the day of the year. The sunrise/sunset hour-angle is calculated as:

(2) $$hourAngle=cos^{-1}\left(-tan\left(L\right)tan\left(\phi \right)\right)$$

where *L* is the latitude. The day length (*D*) is calculated by:

(3) $$D=2*\frac{hourAngle}{15}$$

The day length was computed for each unique latitude value in the grid for each day of the year and averaged monthly to match the vector surveillance temporal resolution. The month of February was treated accordingly to account for leap years.

Finally, the climate features (daily minimum, maximum, and average temperature, total precipitation and relative humidity) were obtained from the NASA Earth Exchange (NEX) Global Daily Downscaled Projections (GDDP) (NEX-GDDP-CMIP6) historical and future projections climate datasets; hereafter referred to as NEX-CMIP6 [34]. A complete list of the nine NEX-CMIP6 downscaled models used in this study is shown in Table 1. The provided spatial resolution matches the vector surveillance regular grid used, whereas monthly averages were calculated for the climate variables to match the temporal resolution.

The list of features created using the LUH2 dataset is shown in Table 2.

### 2.2. Machine Learning

**Figure 2:**
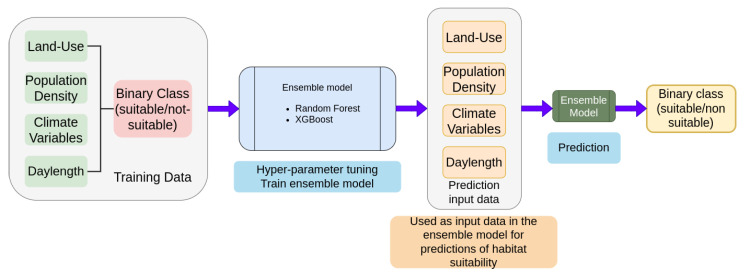
The schematic, high-level overview of the procedures followed in this study to train the machine learning model and project *Ae. albopictus* habitat suitability.

A schematic overview of the training procedures of the machine learning model and projecting *Ae. albopictus* habitat suitability are shown in Figure 2. A binary classification ensemble model, consisting of a Random Forest classifier [27] and an XGBoost classifier [28], was trained using the training dataset described in the relevant section.

The two models were integrated into a single ensemble model, using the VotingClassifier method of the Python scikit-learn package. According to the VotingClassifier method, each model was allowed to perform independent predictions, which were then combined using the Soft Vote method, i.e., the probability for each predicted class is summed and the class with the highest probability sum is chosen,

(4) $$\hat{y}=argmax\left(\frac{1}{N_{classifiers}}\sum_{classifiers}\left(p_{1},p_{2},...p_{n}\right)\right)$$

where $N_{classifiers}$ is the number of classifiers in the ensemble model and $p_{n}$ is the probability assigned to each class by model *n* [54,55].

To train the models and evaluate the performance, the gridded monthly vector presence/absence dataset was randomly partitioned into two parts, training and test sets, following a 90-10 ratio, that is, 90% of the data was used to train each of the two models and the remaining 10% was used for validation. The performance of each model was measured using the *F1-score* metric,

(5) $$F1\;Score=2*\frac{1}{\frac{1}{precision}+\frac{1}{recall}},$$

where,

(6) $$\begin{array}{c} Precision\;\left(P\right)=\frac{True\;Positives}{True\;Positives\;+\;False\;Positives}\;\mathrm{and}\\ Recall\;\left(R\right)=\frac{True\;Positives}{True\;Positives\;+False\;Negatives}. \end{array}$$

In addition, the specificity (True Negative Rate) was used, defined as,

(7) $$\begin{array}{c} Specificity=\frac{True\;Negatives}{True\;Negatives\;+\;False\;Positives} \end{array}$$

The F1-score metric provides a reliable assessment, particularly when the number of positive and negative classes in the training dataset are unbalanced [56]. The metric is evaluated in the range of zero to one, with zero being the lowest (worst) and one the highest (best) achievable score. To train the binary classification models of the ensemble, hyper-parameter tuning was performed using the GridSearchCV method (scikit-learn package of Python) with the F1-score as the performance metric.

As the final performance measure, ensemble predictions were compared with the most recent reports of global *Ae. albopictus* presence. To compose the global presence dataset, the global compendium of reported presence, compiled by Kraemer et al. (2015) [5], was combined with the reported presence records of the ECDC (obtained through a data request to the relevant authority). The records were assembled into two categories (0: unknown or absence, 1: reported presence) and were re-gridded to match the vector presence/absence dataset.

To estimate the ensemble model’s sensitivity, we used an arbitrary threshold of monthly presence, which we applied to the decadal averages of the ensemble’s model output between 2015 and 2025, for both the SSP245 and SSP585 scenarios (average number of months per year predicted as suitable over the specified time period). Each grid cell was compared to a threshold value, which we varied between 0 and 12 months, with the grid cell considered suitable for *Ae. albopictus* establishment if the ensemble model predicted presence for more months than the threshold and was not suitable otherwise. We subsequently compared the obtained habitat suitability maps for each threshold value with the aforementioned global presence dataset to assess the sensitivity of the ensemble model.

### 2.3. Population at Risk

To estimate the total population at risk from *Ae. albopictus*-borne diseases, we estimated the population residing in each grid box using the population density dataset for each year and scenario. For each year, we considered the population that resides in grid boxes where three or more months are predicted as suitable from the ensemble model to be at risk. In their study of the suitability of the European climate for *Ae. albopictus*, Caminade et al. (201) established a threshold of 18 weeks of activity to be considered as suitable, whereas Petric et al. (2021) report this threshold to be as low as 12 weeks [57,58]. We, therefore, considered a middle ground between the two as a limit for risk of 14 weeks or ∼3 months. Historically, vector introduction to new regions has been recorded to be through the transport of dormant mosquitoes in the egg stage through trade or in very limited amounts in the adult stage [6,10,14,59]. It is, therefore, unlikely, that the vector invades a region with limited habitat suitability in adequate numbers in the adult stage to pose significant threat to human health.

To take the global population and the global suitable habitats expansion into consideration, we performed the analysis using the initial habitat range (grid points deemed as suitable for the year 2020 for the SSP245 scenario) as a reference. Next, the median of the population at risk from the six climate models for each scenario was calculated for the periods of 2020–2025, 2045–2055, and 2095–2100, and compared to the reference population at risk.

### 2.4. Maps

All the maps presented in this study were created using the cartopy module in Python 3.9 [60] by making use of the Natural Earth raster and vector map data, which are freely available in the public domain (free vector and raster map data at naturalearthdata.com).

## 3. Results and Discussion

The machine learning model we developed employs a combination of binary classification algorithms, namely, Random Forest and XGBoost classifiers, trained with a collection of global reports of monthly *Ae. albopictus* presence and gridded climatic, land use, and human population datasets. The output of the model is a boolean indicator of habitat suitability, where 1 indicates suitability in a grid cell in a given month and 0 otherwise. Due to the nature of the data used in training this model, in this context, habitat suitability is defined as the grid box for a given month having favorable climatic and environmental conditions for the *Ae. albopictus* mosquito to survive. In this section, we present and discuss the results of the training procedures and the predicted habitat suitability of the vector until the end of the 21st century.

### 3.1. Machine Learning Model

The ML model comprised two independent binary classification models, namely, a Random Forest and an XGBoost classifier. The two binary classification models were trained on a total of 51,000 examples (90% of the feature dataset), whereas approximately 6000 examples were withheld from the feature set to be used a test set. The two models achieved F1-scores of 0.90 and 0.91, respectively, on the test set. The two models were subsequently combined to form an ensemble model, using the VotingClassifier method in the scikit-learn Python module, using the Soft Vote method, in which the prediction with the highest probability sum from the two independent models was chosen for each grid cell/month. The ensemble model achieved an F1-score of 0.92 in the test set and a 10-fold cross-validation yielded an F1-score of 0.89 ± 0.06.

**Figure 3:**
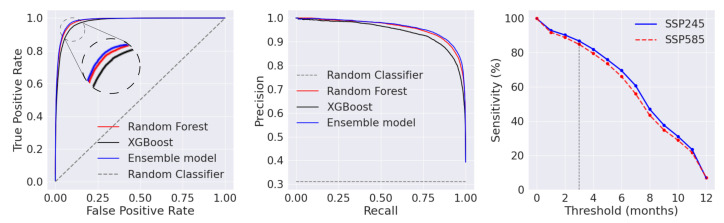
The ROC curves obtained on the test dataset (**left** panel) and precision-recall curves (**middle** panel) for the Random Forest, XGBoost and, ensemble models. On the (**right** panel), the sensitivity of the ensemble model compared to the known presence of *Ae. albopictus* as a function of the number of months set as a threshold for habitat suitability. The inset in the (**left** panel) shows a zoomed view of the ROC curves.

The associated Receiver Operating Characteristic (ROC) curve, precision-recall curve, and the sensitivity compared to the known presence of the vector are shown in Figure 3. The obtained Area under the ROC curve (AUC), an empirical measure of the classification models’ performance, was 0.97, an indication of good classification performance on the test set by the trained model [61]. In addition, the precision-recall curve provides further indication of good performance by the model on the test set, as there is minimal precision to recall trade-off. In both curves, there is a small, albeit measurable, increase in performance in the test set when combining the two independent models into an ensemble model.

A potential limitation of the current study is the restriction of the observational training data to certain regions of Europe and the USA. To assess the global applicability of the machine learning model, we used the global compendium of reported *Ae. albopictus* presence and the ECDC VectorNet database (see Section 2), and calculated the sensitivity as the percentage of grid points where predicted habitat suitability is in agreement with the reported presence. We varied the threshold of annual suitability, i.e., the number of months predicted as suitable required to label a grid cell as suitable, and averaged the annual suitability over the years 2020–2025 for both SSP245 and SSP585 scenarios. We considered a minimum of approximately 3 months (∼14 weeks) of predicted suitable months for a grid cell to sustain an *Ae. albopictus* population for over a year. At this limit, the machine learning model achieved a sensitivity score of ∼86% for both scenarios.

There have been several research articles focusing on developing models for *Ae. albopictus*, employing a wide array of methodologies, such as environmental niche models [25], genetic algorithms [23], mechanistic modeling [62,63], and fuzzy modeling methodologies [18]. In addition, other studies have employed machine learning methods, such as the maximum entropy algorithm [8,64,65,66], boosted regression trees (BGT) [5], and others, including random forest and support vector machine (SVM) models [67].

In the maximum entropy modeling approach, known presence data are used in order to train the model to identify areas of similar conditions and create maps of habitat suitability. Such an approach does not take into consideration temporal fluctuations in the areas, such as the temperature and precipitation within a year, and is not able to predict habitat unsuitability. Ding et al. (2018) used a Random Forest classifier to outperform other algorithms, such as the SVM and GBT, in mapping the potential spatial distribution of *Ae. aegypti* and *Ae. albopictus*. These models were trained using known presence data though and were not used to make predictions into the future [67]. Fruh et al. (2018) compared four machine learning models in classifying *Ae. japonicus* occurrence in Germany, and have found that climatic predictors on their own were not able to adequately train the models; further aspects were necessary, such as land use and host population density [68], which have been included in this study.

The feature set selection in this study aimed to characterize the spatio-temporal grid using both climatic/environmental variables and add human-driven aspects, such as population density and land use. The feature selection was based on the biological and environmental dependencies of the mosquito life cycle, as temperature, precipitation, and relative humidity directly affect the reproduction, development, and the survival of *Ae. albopictus* [69]. Day length has been demonstrated to also affect the life cycle of the mosquito [70]. *Ae. albopictus* has been observed to feed from a variety of hosts, including humans, domestic and wild animals, reptiles, birds, and amphibians, even though a preference towards human hosts has been documented [71,72]. The vector has also been observed in forested areas, close to the urban/forest interface [73]. To satisfy these vector dependencies to human-driven aspects, we have included human population density and related land use features, which were created from combining the associated classes in the LUH2 land use dataset, i.e., potential forested/non-forested, urban, crops and graze land. The inclusion of the non-forested class was necessary, as the model was trained to predict both habitat suitability and non-suitability.

In addition, a significant advantage of our proposed approach is the use of longitudinal data in training the model and projecting habitat suitability in the future. This can allow the model to capture the dependency of *Ae. albopictus* habitat suitability on short-term fluctuations, e.g., how temperature and precipitation fluctuates in a given grid cell within a year, and long-term fluctuations, such as the effects of the growing human population or the increase in urban land use in a grid cell.

### 3.2. Habitat Suitability under Climate Change

**Figure 4:**
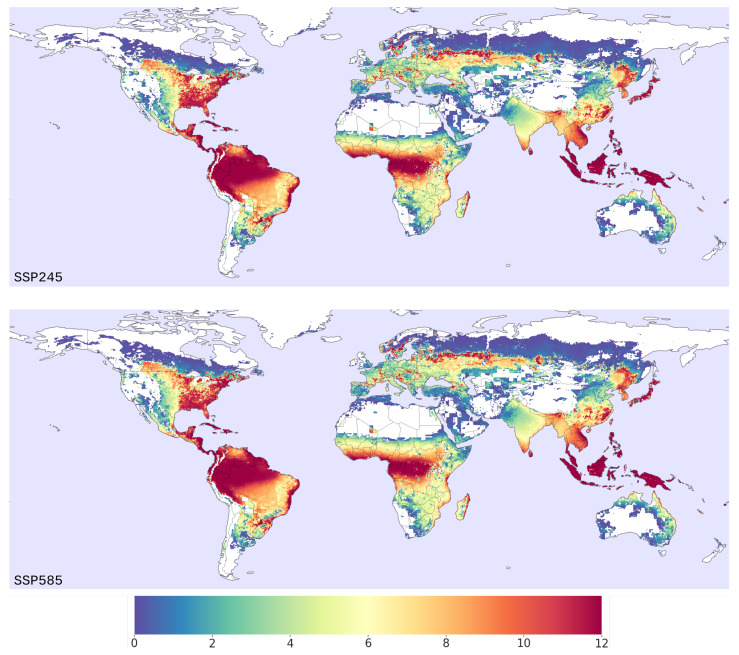
Predicted *Ae. albopictus* habitat suitability in terms of months predicted as suitable by the ML model for the early part of the century (2020–2025). The normalization scenario (SSP245) is presented on the top panel, whereas the “business as usual” (SSP585) scenario is presented on the bottom panel. The colorbar shows the average number of months predicted as suitable by the machine learning model for each grid cell.

The average output of the nine climate models for the two climate scenarios (SSP245 and SSP585) of the ML model for *Ae. albopictus* habitat suitability in terms of total months predicted as suitable for the early part of the century (2020–2025 ensemble average) is shown in Figure 4. Parts of the world such as the USA’s eastern coast, central Africa, eastern parts of Asia and the northern regions of South America are predicted to be able to host *Ae. albopictus* populations for the majority of the year. In Europe and Australia, the predicted habitat suitability is less severe.

Direct comparison of the predicted global distribution of habitat suitability with other published studies for the same vector is difficult due to the different outputs of the models and the use of the newly released CMIP6 climate projections. The habitat suitability maps in this study most closely resemble the projected distribution published by Kamal et al., (2018) with the most notable difference being the extension of habitat suitability towards Russia and central North America in this model [25]. Compared to other similar studies, which utilised BRT and ENM approaches, the machine learning estimates higher habitat suitability in the Northern hemisphere, such as in northern Europe and the central parts of North America, but closely resembles the maps generated for the Southern hemisphere [4,18,57,65,74].

It should be noted that we do not distinguish between the different strains of *Ae. albopictus*, but the majority of data used to train the machine learning model originates from North America, where the temperate strain, which has the ability to overwinter [75], have been extensively observed. It is, thus, logical to assume that the climate, land use, and population relations with respect to habitat suitability learned by the model mostly reflects that of the temperate strain of the mosquito and are a potential explanation of the higher degree of suitability predicted by the model in northern areas of the world. In addition, the model treats each month independently, with no temporal dependencies.

**Figure 5:**
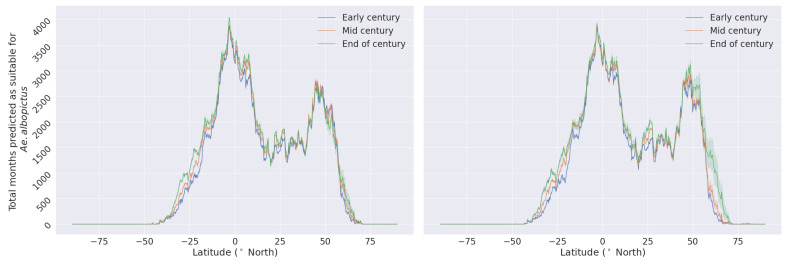
Comparison of the latitude profiles for the SSP245 climate scenario (**left** panel) and SSP585 (**right** panel), for the early, mid, and end of century time periods. Solid lines and the shaded areas represent the median and the 95% range, respectively.

**Figure 6:**
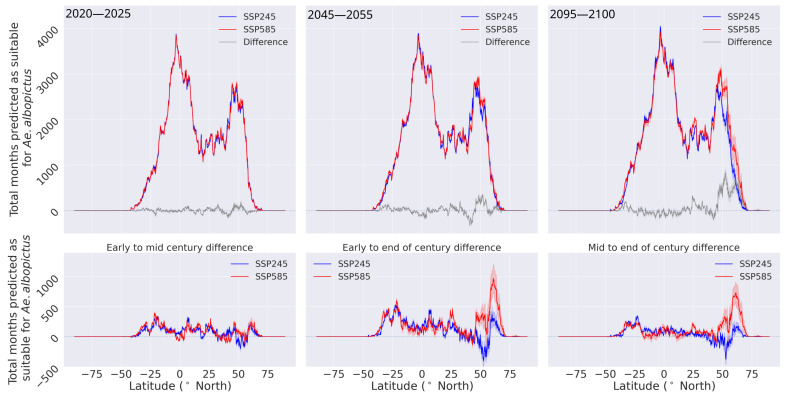
Latitude profiles for the total number of months predicted as suitable by the ML model (**top** panels) and the transitional changes between the early to mid, end of century, and mid to end of century periods (**bottom** panels). The latitude profiles obtained for the SSP245 scenario are shown in red and the corresponding profiles for the SSP585 scenario are shown in blue, whereas the difference between the two is shown in gray. Solid lines and the shaded areas represent the median and the 95% range, respectively.

Prompted by the observed level of applicability, we projected the global impact of climate change on *Ae. albopictus* habitat suitability until the end of the century. To investigate its effects’ on habitats and global distribution, we calculated the latitude profiles by summing the total number of predicted months for each year per latitude. Furthermore, we calculated the average of three time periods in the 21st century, early century (2020–2025), mid century (2045–2055) and end of century (2095–2100). The predicted latitude profiles for the three time periods and the transitional differences between the early to mid, early to end, and mid to end of century time periods are shown in Figure 5 and Figure 6.

In both climate scenarios, there is an increase in the number of months predicted as suitable for *Ae. albopictus* across all the latitudes throughout the 21st century, most notably in the two extremities of the distribution. This indicates a polewards expansion of suitable habitats, as a result of climate and land use changes until the end of the century. Comparing the projected suitable habitats expansion to the literature, similar polewards extension is predicted by various previously published studies [5,18,57,74].

In the early part of the century, the two climate scenarios predict nearly identical global distribution of *Ae. albopictus* habitat suitability, whereas after the mid-century time period, the two start to diverge, especially in the Northern hemisphere. In the mid-century period, there is minimal difference between the two scenarios in the Southern hemisphere, in contrast to the Northern, where the prediction produced by the SSP245 scenario dominates around the 50${}^{\circ }$ N latitude and the SSP585 dominates above this. Towards the end of the century, the two scenarios have similar latitude profiles up to 50${}^{\circ }$ N. In latitudes above 50${}^{\circ }$ N, in the SSP585 scenario there is significantly higher number of suitable months predicted as suitable compared to the SSP245 scenario. These differences can also be observed in the transitional graphs between the time periods (bottom row of panels, Figure 6). In the early to mid-century period transition, both scenarios have similar changes. The most notable transitions are observed in the second part of the century for the SSP585 scenario, as above 50${}^{\circ }$ N there is a large increase in months predicted as suitable.

**Figure 7:**
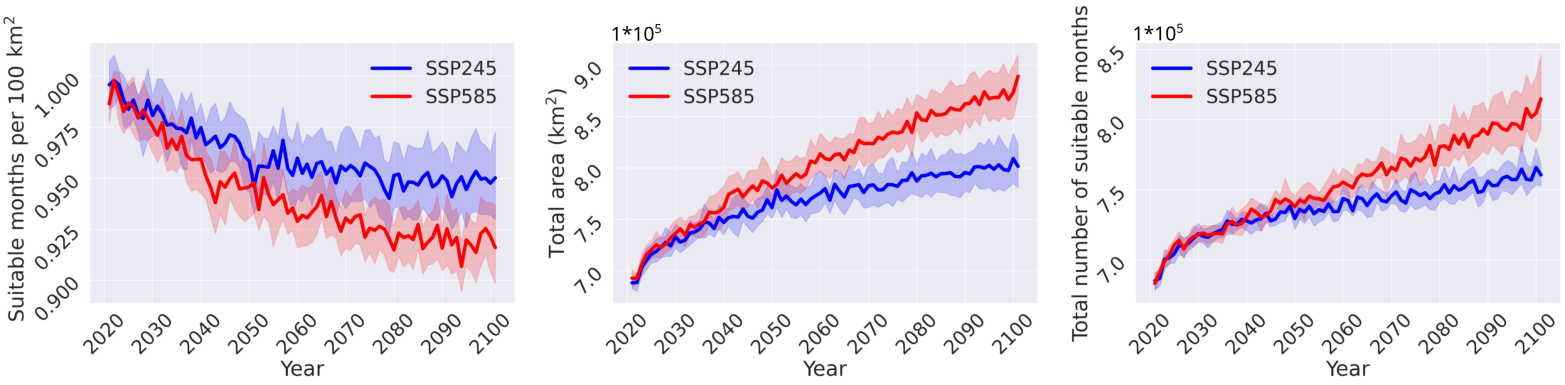
Suitable months normalised to 100 km${}^{2}$ (**left** panel), the total area over which habitat suitability is projected (**middle** panel) and the total number of months projected as suitable for *Ae. albopictus* (**right** panel), for the two IPCC scenarios. The blue and red lines, which correspond to the SSP245 and SSP585 scenarios, respectively, show the ensemble average for the nine climate models used for each of the two scenarios. Solid lines and the shaded areas represent the median and the 95% range, respectively.

The two IPCC emission scenarios used in this study follow a similar trend in temperature until about 2030, after which growth under the SSP245 scenario weakens, whereas under the SSP585 scenario growth continues to follow a steep increase [76]. A similar trend is also evident in the total number of projected suitable months per year, shown in Figure 7. The projections for the two scenarios follow a similar trend until around the year 2040, after which they diverge. The rate of growth for the SSP585 scenario is largely unchanged throughout the century, whereas for the SSP245 its significantly reduced. Similarly, the total area over which at least one month is projected as suitable follows a near identical trend. Interestingly, normalizing the suitable months to area, in this case, 100 km${}^{2}$, follows the opposite trend. In both scenarios, the curves follow negative growth trend; the SSP245 scenarios seems to reach a relatively steady state in the second part of the century, whereas the SSP585 scenario continues to decrease until the end of the century.

**Figure 8:**
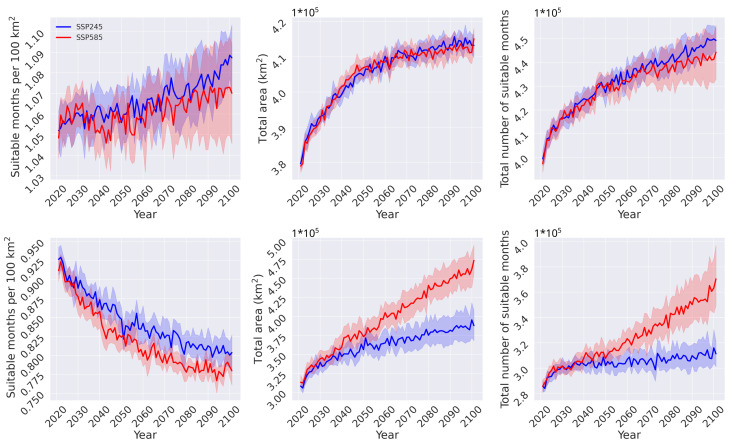
Comparison between the tropical (**top** row of panels) and extratropical (**bottom** row of panels) regions of the world for habitat suitability normalised to 100 km${}^{2}$ (**left** column), total area covered (**middle** column) and total number of months predicted for each year (**right** column). Solid lines and the shaded areas represent the median and the 95% range, respectively.

To further investigate the geographical shifts of *Ae. albopictus* habitat suitability, we subdivided the projections with respect to latitude, i.e., into the tropical and extra-tropical regions of the world (Figure 8). The tropical latitude range is defined by −23.5${}^{\circ }$ to 23.5${}^{\circ }$ latitude (south and north, respectively), and the extra-tropics are the regions that lie poleward of this range. In the tropics, the two scenarios follow nearly identical trends. Both scenarios show a significant increase in total number of projected suitable months and total area with at least one month projected as suitable. Both grow at a similar rate, which is reflected in the normalized suitable months per 100 km${}^{2}$ curve. In the extra-tropical regions, the SSP245 scenario has an initial growth phase in the total number of projected suitable months until around the year 2030, after which it plateaus. The SSP585 scenario, follows a similar trend until year 2030, after which it follows a monotonic increasing trend until the end of the century. In the total area curves, both scenarios follow a similar increasing trend until the year 2030, after which the rate of growth of the SSP245 is significantly reduced, whereas for the SSP585 it continues to grow until the end of the century. Lastly, there is a net decrease in both scenarios for the suitable months per 100 km${}^{2}$, as a result of the higher area expansion compared to suitable months. Based on these results, the habitat suitability for *Ae. albopictus* will remain relatively constant in the tropical regions, whereas there will be a net decrease in the extra-tropical regions, since the area in which at least one month is predicted as suitable is projected to grow at a higher degree compared to the total number of suitable months.

**Figure 9:**
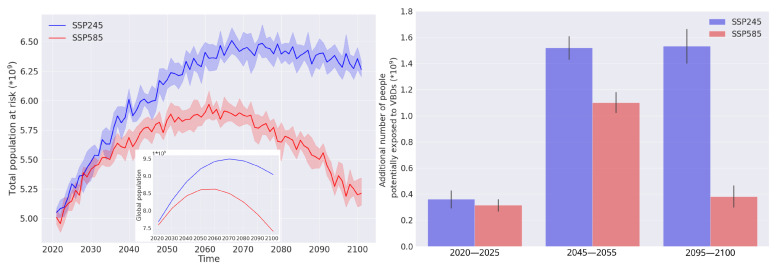
Population at risk of *Ae. albopictus*-borne diseases per year for the two scenarios examined in this study (**left** panel). In the inset, the total population projected until the end of the century for the scenarios is presented. In addition, the increase in population at risk of *Ae. albopictus*-borne diseases with respect to the start of the projection window (2020) for the SSP245 scenario (blue) and SSP585 scenario (red) is shown on the right panel. The three time periods presented here correspond to the early, mid, and end of the 21st century. The median of the output from the nine climate scenarios is presented here and the shaded area (**left** panel) and lines (**right** panel) represent the 95% range.

Using the ensemble of the nine climate models for the SSP245 and SSP585 scenarios, approximately five billion people are predicted to inhabit areas suitable for the establishment of *Ae. albopictus* in the year 2020, as shown in Figure 9. Grid cells in which three or more months are predicted as suitable by the machine learning model were considered to be able to sustain an established population of the vector (see Section 2). This is about 1.5 billion higher than the best estimate by Proestos et al. (2015), but matches the population at risk of dengue predicted by Messina et al. (2019) [18,77]. The projected population at risk for both scenarios peaks around the year 2060, driven by the geographical expansion of suitable habitats and population growth. In the SSP245 scenario, the population at risk curve reaches a plateau after that point, whereas in the SSP585 scenario, there curve follows a negative growth trend.

We projected a dramatic increase in the number of people at risk for the mid-century time period (2045–2055) for both IPCC scenarios (up to 1.5 billion for SSP245 and 1.1 for SSP585). We found that SSP585 consistently leads to a more moderate increase, which manifests into a steep difference between the two scenarios for the end-of-century time period (2095–2100). This striking difference is mainly attributed to the projected adverse effect of the business-as-usual scenario on human population at the end of the 21st century [42]. Even though the total population in the SSP585 scenario is projected to fall below the current level, an additional 0.4 billion people are predicted to be at risk of *Ae. albopictus*-borne diseases by the end of century, as a result of suitable habitat expansion.

The surveillance data used in the training and test sets were pooled from various sources, as described in the Section 2. They include data collected using various surveillance methodologies, such as ovitraps, larvae collection, and adult traps. To optimize costs and human resources, many surveillance schemes do not operate year-round, but rather concentrate around the peak seasons or operate at a significantly reduced capacity outside of it; thus, the negative points were possibly under-represented or missing. This lead to the exclusion of certain grid cells, as it could potentially add unwanted bias to the trained model. Furthermore, data from regions where *Ae. albopictus* is known to have well-established populations, such as Brazil, central Africa, and South East Asia, were not available. The limited availability of data and the fact that most of the surveillance data originate from the USA, where the temperate strain of the mosquito is prevalent, potentially restricts the spatio-temporal applicability of the machine learning model. The impact of this limitation on model applicability was low, as confirmed by the model performance and agreement with the global presence data, but was not negligible. Our model predictions largely agree with other models in the Southern hemisphere, but predict significantly larger habitat suitability in the Northern hemisphere, especially in south west Russia [18,25,65]. Comparing the projected suitable habitats expansion to the literature, similar polewards expansion is also predicted by the vast majority of published studies [18,57,74,77].

The strong dependence of the machine learning methodologies on the extent, quality, and availability of the vector surveillance data should be emphasized. There is a need for a centralized database in which reliable surveillance data can be shared between researchers. Access to a comprehensive dataset will allow researchers to develop high-resolution models that can learn temporal dependencies, such as long-short term memory (LSTM) neural networks, which can support more effective surveillance and early detection policies in the future. Such a database can allow researchers to take advantage of the rapid advancements in data science and infrastructure to develop models and services for *Ae. albopictus* and other important vectors of human diseases.

## 4. Conclusions

In this study we used climate, population, and land use features to spatio-temporally characterize a gridded area where field surveillance data were available for the invasive *Ae. albopictus* mosquito species, a vector of potentially deadly arboviruses. This was subsequently used to train a binary classification machine learning model to predict habitat suitability for the vector. The machine learning model was then used to project the habitat suitability on a global scale until the end of the 21st century and to assess the impact of climate change for two IPCC scenarios, SSP245 and SSP585, aimed at climate change stabilization and business-as-usual growth, respectively.

The two scenarios do not deviate significantly until the year 2030 in terms of the total number of predicted suitable months for *Ae. albopictus* and the total area in which a suitable habitat is predicted. After that and until the end of the century, the SSP245 rate of growth is significantly reduced, whereas the SSP585 scenario continues to grow. In both scenarios, a polewards expansion of habitat suitability is projected, which can expose hundreds of millions of people to *Ae. albopictus*-borne diseases.

Our results suggest that municipal administrations and concerned parties in nations on the cusp of becoming suitable to the establishment of *Ae. albopictus* should be poised to implement preventative measures through coordinated surveillance initiatives at potential ingress points, such as harbors, airports, and commercial routes. Moreover, it is imperative that public health authorities in the aforementioned nations implement targeted intervention strategies to mitigate the dissemination of vector-borne diseases.

In conclusion, we have implemented a machine learning approach to vector borne disease modeling and our findings suggest that climate change can play a significant role in the poleward expansion of *Ae. albopictus*. This may pose challenges to areas of the world where vector populations are currently not present and add to the challenges in areas that already face problems with vector-borne diseases.

## Figures and Tables

**Table 1:** List of the NEX-CMIP6 global downscaled climate models used in this study.

Name	Long Name	Ref.
ACCESS-ESM1-5	Australian Community Climate and Earth System Simulator (ACCESS)	[45]
EC-Earth3	EC-Earth European Consortium	[46]
GFDL-CM4	Geophysical Fluid Dynamics Laboratory (GFDL)	[47]
FGOALS-g3	Flexible Global Ocean-Atmosphere-Land System Model Grid Point Version 3	[48]
INM-CM4-8	Institute of Numerical Mathematics (INM)	[49]
INM-CM5-0	Institute of Numerical Mathematics (INM)	[50]
MIROC6	Model for Interdisciplinary Research on Climate	[51]
MRI-ESM2-0	Meteorological Research Institute Earth System Model Version 2.0	[52]
NorESM2-MM	Norwegian Earth System Model	[53]

**Table 2:** Feature set used in training the supervised machine learning models and projecting *Ae. albopictus* habitat suitability.

Name	Long Name	Units
tas	Average temperature	${}^{\circ }$C
tasmin	Minimum temperature	${}^{\circ }$C
tasmax	Maximum temperature	${}^{\circ }$C
tp	Total precipitation	mm
hurs	Relative humidity	%
pop_density	Population density	per sq. km
daylength	Day length	hours
urban	Urban land use	Fraction coverage
crops	Crops related land use	Fraction coverage
forested	Potential forest land use	Fraction coverage
non-forested	Potential non-forest land use	Fraction coverage
graze-land	Grazing land use	Fraction coverage

## Data Availability

The data used in this study are freely available and cited appropriately.

## Abbreviations

The following abbreviations are used in this manuscript:

CMIP6	Coupled model intercomparison project 6
RCP	Representative concentration scenario
SSP	Shared socioeconomic pathway
ENM	Environmental niche model
BRT	Boost regression trees
SVM	Support vector machine
LUH2	Land use harmonization 2
NEX	Nasa earth exchange
GDDP	Global daily downscaled projections
ECDC	European centre for disease control
XGBoost	Extreme gradient boost
ROC	Receiver operating characteristic
AUC	Area under curve
ML	Machine learning
LSTM	Long short-term memory
